# Biochemical basis of cervical remodeling in preterm and term labor revealed by Raman spectroscopy

**DOI:** 10.1002/btm2.70193

**Published:** 2026-11-10

**Authors:** Rekha Gautam, Christine M. O'Brien, Ezekiel J. Haugen, Jackson H. Rogers, Laura E. Butler, Jennifer N. Bateman, Anna S. Rourke‐Funderburg, Naoko Boatwright, Wilson R. Adams, Giju Thomas, Jeff Reese, Jennifer L. Herington, Anita Mahadevan‐Jansen

**Affiliations:** ^1^ Vanderbilt Biophotonics Center Vanderbilt University Nashville Tennessee USA; ^2^ Department of Biomedical Engineering Vanderbilt University Nashville Tennessee USA; ^3^ Biophotonics@Tyndall, IPIC, Tyndall National Institute Cork Ireland; ^4^ Department of Biomedical Engineering Washington University in St. Louis St. Louis Missouri USA; ^5^ Department of Pediatrics Vanderbilt University Medical Center Nashville Tennessee USA

**Keywords:** cervix, lipopolysaccharide, mifepristone, preterm labor, Raman spectroscopy, second‐harmonic generation, two‐photon excitation fluorescence

## Abstract

Preterm birth (PTB), affecting approximately 15 million infants annually, is the leading cause of neonatal mortality. However, the underlying mechanisms driving preterm labor (PTL) remain poorly understood. Early detection of biomolecular changes associated with both term and PTL is critical but challenging with current methods. Here, we utilize in vivo Raman spectroscopy (RS) to non‐invasively investigate cervical remodeling in mouse models of PTL induced by inflammatory (lipopolysaccharide ‘LPS’) and hormonal (RU486) pathways, compared with term labor (TL). In vivo RS results revealed etiology‐specific biochemical differences associated with cervical remodeling. LPS‐induced PTL exhibited increased carbohydrate‐associated spectral features, whereas the RU486 model exhibited a distinct protein remodeling pattern that diverged from both LPS‐induced PTL and TL. These biochemical signatures aligned with ex vivo imaging and biomechanical assessments of collagen, elastin, and vascularity. RS differentiated PTL from TL with 86% accuracy, outperforming existing techniques. Collectively, these results demonstrate that PTL is not a single biochemical process but comprises distinct remodeling trajectories that can be detected in vivo prior to labor onset. This work establishes a translational framework for Raman‐based cervical phenotyping, supporting the future development of non‐invasive tools for early PTL detection, etiological stratification, and targeted clinical intervention, to improve maternal and neonatal outcomes.

## Graphical abstract

In vivo Raman spectroscopy enables non‐invasive biochemical phenotyping of distinct preterm cervical remodeling pathways, with Raman biomarkers validated against biomechanics, imaging, and histology to support future clinical translation.

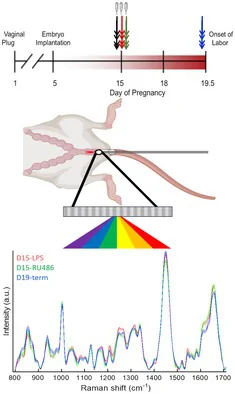

**Translational Impact Statement**

This work demonstrates the translational potential of in vivo Raman spectroscopy (RS) as a non‐invasive tool to characterize distinct cervical remodeling mechanisms associated with PTL. By capturing biochemical changes prior to structural failure, RS differentiates preterm from term labor with 86% accuracy, exceeding current clinical assessments. Integration of in vivo data with ex vivo imaging and biomechanical analysis reveals etiology‐specific remodeling signatures. These findings support the use of in vivo RS for longitudinal monitoring to identify high‐risk individuals early, enabling timely intervention and improved neonatal outcomes.

## 1 INTRODUCTION

Preterm birth (PTB), defined as delivery before 37 weeks of pregnancy, affected 4–16% of pregnancies (~13.4 million) in 2020. Alarmingly, PTB accounted for ~16% of deaths in children under age five and 35% of neonatal mortality.^1^, ^2^ Factors contributing to spontaneous preterm labor (PTL) include uterine overdistension, inflammation with/without infection, stress, hemorrhage, cervical insufficiency, genetics, smoking, and substance abuse.^3^, ^4^ Although the etiology of PTL is complex, research has unveiled that many of these factors induce molecular changes in the cervix.^5^, ^6^, ^7^

The cervix acts as a mechanical barrier to maintain the fetus in the uterus during pregnancy and undergoes dynamic remodeling essential for parturition. This cervical remodeling occurs in overlapping phases (softening, ripening, dilation, postpartum repair) and involves changes in extracellular matrix (ECM) proteins, glycans, lipids, vascular perfusion, and hydration.^8^ Cellular and ECM protein organization critically influence cervical length, elasticity, and dilation.^7^ Importantly, biochemical changes in the cervix occur well before shortening and softening are detectable by clinical examination.^9^, ^10^ Detecting these changes associated with premature cervical ripening, though vital for the early identification of high‐risk patients, remains a challenging task. Current assessment methods (e.g., Bishop score, cervical length ultrasound) show considerable variability, complicating labor prediction, especially in women with a short cervix at risk of preterm delivery. The integration of PTL biomarkers (e.g., fetal fibronectin in vaginal discharge) enhances diagnostic accuracy but only identifies at‐risk women effectively ~50% of the time.^11^

To address these limitations, optical tools have been studied to non‐invasively assess cervical microstructures and biochemistry. Techniques such as second‐harmonic generation (SHG) visualize collagen structure; optical coherence tomography provides insights into tissue microstructure, akin to ultrasound; absorption and photoacoustic spectroscopy offer molecular specificity; autofluorescence is sensitive to collagen crosslinks; and polarization imaging quantifies ECM alignment and dispersion.^7^, ^12^ However, most of these techniques probe one component at a time, limiting their utility for understanding mechanisms of cervical remodeling.

Raman spectroscopy (RS) measures inelastic light scattering, in which energy is exchanged between an incident photon and a molecule, depending on the molecular structure and bond types present. The relative quantity of a given molecular bond is observed as a proportionate peak in the Raman spectrum, and the unique energy shift of a peak reflects the vibrational energy of that particular bond. Together, energy shifts and peak heights represent the biochemical makeup of the sample. RS allows simultaneous monitoring of a wide range of biochemicals including lipids, ECM proteins, blood, and glycans.^13^, ^14^ Recently, in vivo Raman studies have provided real‐time information on the biochemical changes that occur in the cervix throughout normal, healthy pregnancies.^8^, ^15^

Given the multifactorial etiology of PTL, mouse models are widely used to elucidate cervical remodeling mechanisms associated with different PTL pathways.^16^, ^17^, ^18^, ^19^ Two commonly used models of induced PTL involve lipopolysaccharide (LPS) to elicit a proinflammatory response or mifepristone (RU486) to induce progesterone (P4) withdrawal.^16^, ^17^, ^18^, ^20^ In LPS‐treated mice, cervical changes include immune cell infiltration, upregulation of proinflammatory genes, cytokines, and matrix metalloproteinases (MMPs).^16^, ^17^, ^18^, ^20^, ^21^ Notably, P4 levels remain high throughout infection/inflammation‐induced labor, and collagen and water content do not change significantly,^18^, ^20^, ^21^ suggesting that inflammatory activation alone is sufficient for infection‐induced PTL but not required for term cervical ripening.^17^ In contrast, RU486‐treated mice exhibit features of accelerated term labor, including reduced P4 levels, increased wet tissue weight, elevated monocytes and eosinophils, and partial activation of inflammatory pathways.^18^ These distinct pathways reflect the heterogeneity of PTL and underscore the utility of mouse models in dissecting PTL mechanisms. However, most cervical data in PTB mouse models have been derived from post‐mortem tissues.

In this study, in vivo RS was used to investigate cervical remodeling in LPS‐ and RU486‐induced PTB compared with term labor in untreated control mice. We hypothesized that temporal changes in cervical biochemical composition, including ECM proteins, lipids, blood, nucleic acids, and carbohydrates, define labor onset and vary by PTL etiology. Distinct Raman spectral changes were observed across term and preterm models. These specific changes were confirmed with ex vivo imaging and biomechanical testing. Overall, these results demonstrate that RS enables non‐invasive, in vivo monitoring of dynamic cervical remodeling, supporting future diagnostic studies in women and early intervention strategies to improve reproductive and neonatal outcomes.

## 2 RESULTS

### 2.1 Significant Raman spectral changes occur during preterm and term cervical remodeling

In vivo transvaginal Raman spectra were acquired using a customized ball lens fiber‐optic probe coupled to a portable RS system (Figure 1a) from the cervix of pregnant mice at day 15 (D15‐control; PBS‐treated, not in labor), induced PTL (D15‐LPS and D15‐RU486), and untreated term labor mice (D19‐term) (Figure 1b). The ball lens probe was designed to minimize interference from surrounding adipose tissue while maintaining sufficient Raman collection efficiency for the small mouse cervix. The resulting penetration depth (~500 μm) enabled preferential interrogation of cervical tissue while avoiding substantial signal contamination from adjacent adipose tissue (Figure S1). In vivo spectral acquisition was non‐destructive, enabling longitudinal monitoring throughout labor.

**FIGURE 1:**
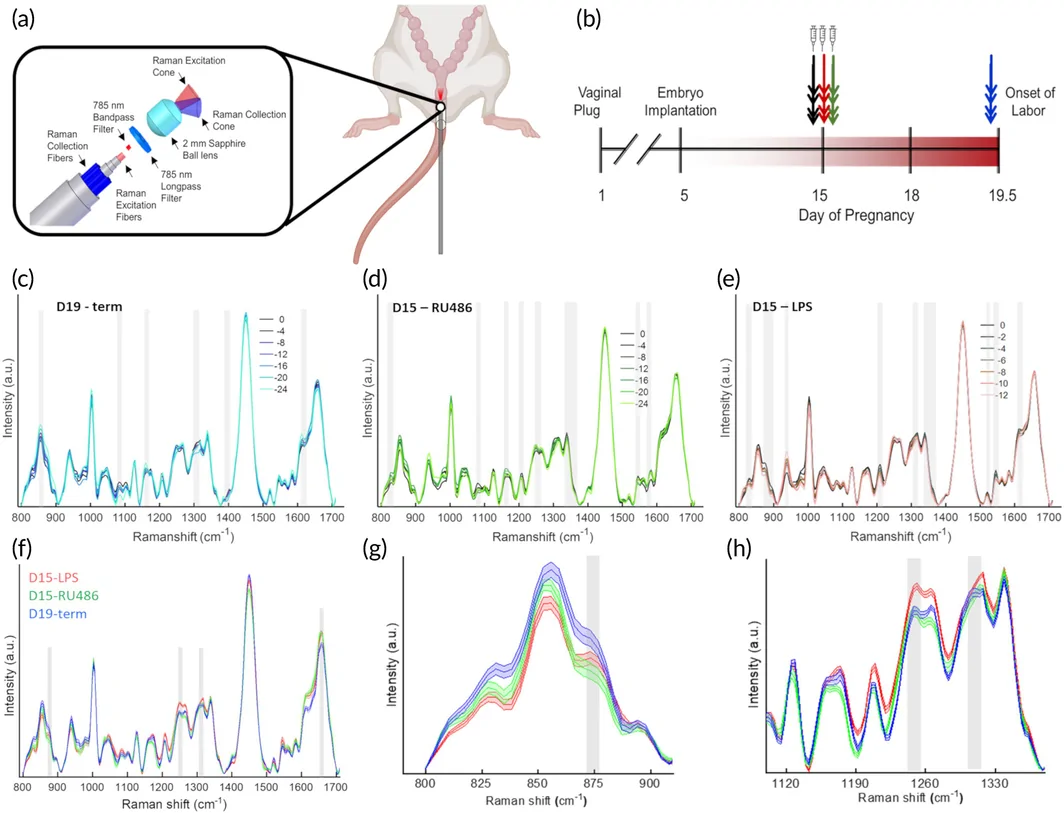
Raman spectral changes observed. (a) Schematic of in vivo Raman spectroscopy ball lens probe for monitoring cervix remodeling during term and PTL in mice. (b) Illustration of the study design for in vivo Raman measurements. RU486 (green) or LPS (red) were administered i.p. to mice on gestation day 15 (D15; morning) to induce preterm birth. Gestation D15‐control mice (black) received PBS. Term labor is labeled with blue. (c–e) are the mean Raman representative spectra plotted as hours prior to delivery over the course of the labor for term D19, RU486‐treated D15, and LPS‐treated D15 mice, respectively. Spectral signatures that differed significantly (*p* < 0.05) with the progression of labor are denoted by vertical gray bands, (*n* = 10–12 per cohort). (f) In vivo 2–4 h prior to delivery Raman spectra for term and preterm groups (D15‐LPS, D15‐RU486, and D19‐term) plotted with the mean and standard error. Spectral signatures that differed significantly (*p* < 0.05) between groups at this time point are marked with vertical gray bands. Magnified spectral region (g) (800–900 cm^−1^) with main contribution from ECM protein skeletal (C‐C) modes and (h) (1140–1380 cm^−1^) mainly show the amide III region associated with ECM protein structural changes.

LPS‐ and RU486‐treated mice on D15 exhibited labor approximately 8 and 16 h post administration, respectively, consistent with prior intrauterine pressure measurements.^22^ In this study, mean delivery times for LPS‐ and RU486‐treated PTL mice differed significantly (11.7 vs. 22.3 h post injection, *p* < 0.05), and both occurred earlier than delivery in untreated D19 term mice. To assess spectral trends as delivery approached, mean Raman spectra were plotted as a function of hours prior to delivery for term and PTL models (Figure 1c–e). These plots illustrate progressive spectral changes associated with cervical remodeling and are relevant to human labor.^8^, ^15^ Raman band assignments, based on prior literature and isolated molecule spectra (Figure S2), are provided in Table S1.^13^, ^14^, ^23^, ^24^, ^25^, ^26^, ^27^, ^28^, ^29^, ^30^, ^31^ Mean spectra acquired 2–4 h before delivery showed significant differences between groups (*p* < 0.05) (Figure 1f–h). Raman band intensity ratios were also calculated and plotted as a function of hours until delivery for all labor models and controls to assess temporal trends, with key spectral markers discussed below.

### 2.2 Conformational changes in ECM proteins segregate PTL from TL models

The RS intensity ratio of 817 cm^−1^/855 cm^−1^ is associated with protein structural changes in the tissue.^13^, ^25^, ^26^ A significant decrease at 817 cm^−1^ (C‐C skeletal mode of proteins) in PTL models indicates that the cervices are under strain (Figure 2a). The 855 cm^−1^ indicates total protein and is assigned to tyrosine fermi doublet, amino‐acid side‐chain vibrations of proline, and CC‐H deformation (aromatic side chains). These changes were specific to the PTL models, as the trajectories for the observation time of 12 h in D15 (untreated control) mice were unaffected. Notably, similar trends were observed in ex vivo biomechanics data where the total dilation (initial dilation + displacement at failure during stress‐relaxation testing) in PTL models was significantly greater from D15‐control cervical tissues (Figure 2c). On D19 of pregnancy, the term cervix exhibited the greatest total dilation among all groups. The RU486‐ and LPS‐treated cervices exhibited significantly more dilation than D15‐control as they remodeled to a caliber that enabled passage of a fetus, effectively “catching up” with D19‐term labor dilation.

**FIGURE 2:**
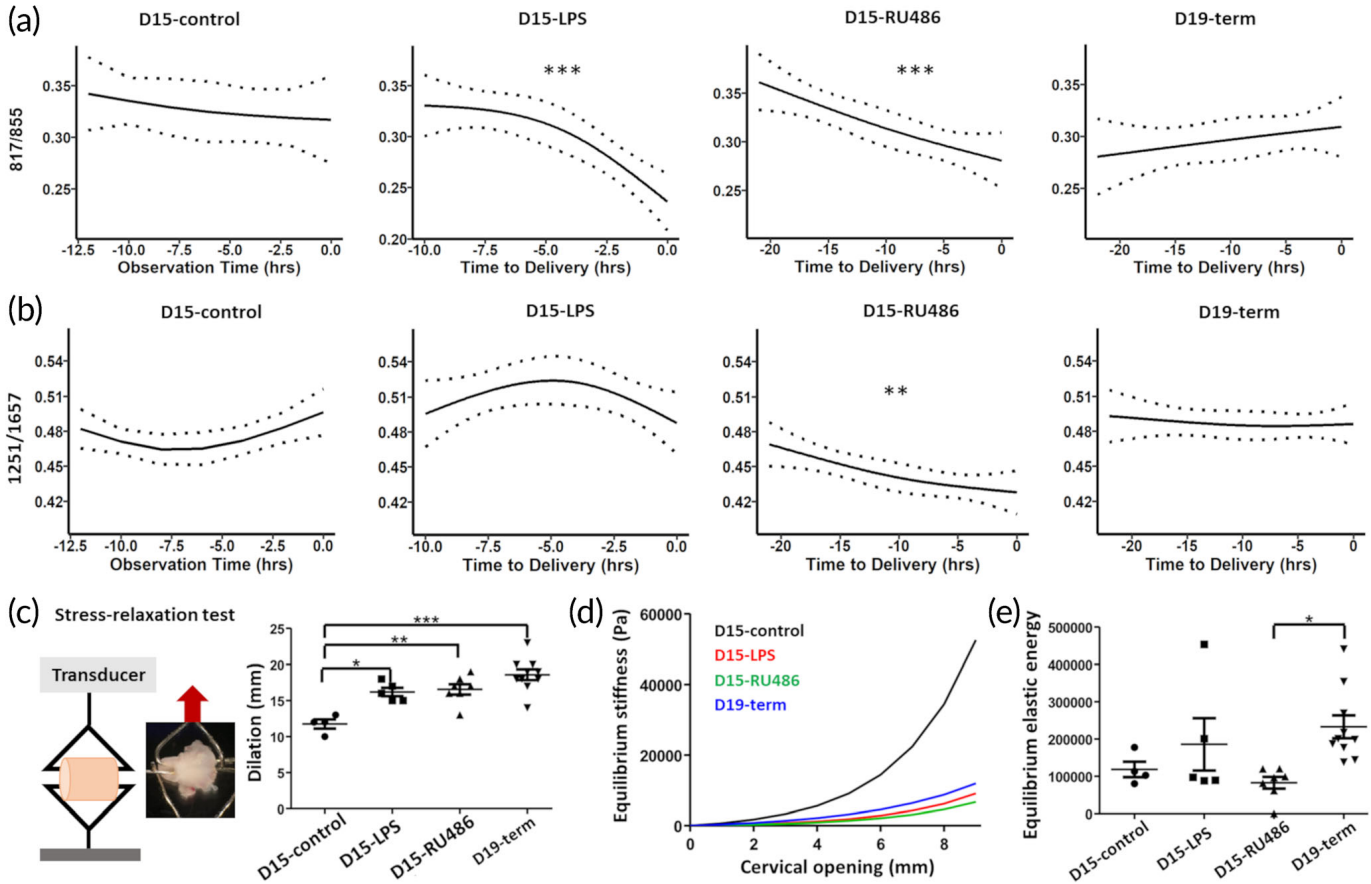
In vivo Raman spectral changes associated with ECM proteins distinguish preterm labor (PTL) from term labor (TL). Longitudinal labor trajectory until delivery (0 h) at (a) 817 cm^−1^/855 cm^−1^ and (b) 1251 cm^−1^/1657 cm^−1^ plotted for four groups—D15‐control, D15‐LPS, D15‐RU486, and D19‐term (left to right)—with mean 95% ± confidence intervals (**p < 0.01, ***p < 0.001 significant change assessed by ANOVA) in slope of the trajectory with respect to horizontal (slope = 0 line) (*n* = 10–12 per cohort) and non‐significant changes left blank. D15‐control trajectory for 12 h was unchanged, as expected. Significant decrease at 817 cm^−1^ (C‐C skeletal mode of proteins) for PTL models reveals that the cervices are under increasingly high load (stress). The amide III/I ratio indicates structural deterioration and loss of ECM proteins integrity isolating D15‐RU486 from other PTL and TL. (c) Ex vivo biomechanical analysis on the mouse cervix samples corroborated the in vivo findings where the total dilation for PTL models was significantly different compared with D15‐control cervical tissues, *n* = 8 per cohort (*p < 0.05, **p < 0.01, ***p < 0.001). (d) Ex vivo biomechanical analysis of cervix samples (*n* = 8 per cohort) using exponential curve fitting of equilibrium stiffness shows lower stiffness in labor groups, with D15‐RU486 cervices having the minimum stiffness observed. (e) Equilibrium elastic energy levels were lowest in the RU486 mice, which could be attributed to specific protein structural changes in RU486 as observed in vivo RS (1251/1657).

### 2.3 Structural changes in ECM proteins separate RU486 from LPS models

The 1251 cm^−1^/1657 cm^−1^ ratio is assigned to amide III/I ratio of ECM proteins. The amide I mode is primarily due to C=O stretching, and amide III is the combination of N‐H bending and C‐N stretching.^31^ These amide modes are sensitive to the secondary structure and orientation of protein fibers including fibrillar collagen.^31^ This ratio significantly decreased over time in RU486 mice, revealing structural changes in ECM proteins specific to RU486 (Figure 2b). RU486 cervices were more compliant than D15 negative control cervical tissues and had values lower than D19‐term (Figure 2d). Further, the elastic energy, calculated as integral stiffness curve to yield point, showed that RU486 cervices stored significantly less energy before deformation than D19‐term, unlike LPS models (Figure 2e). This could be attributed to specific protein structural changes in RU486 mice, indicating greater ECM protein degradation as observed via in vivo RS.

### 2.4 Increase in blood signals during preterm labor catch‐up with term

The 1209 cm^−1^/1275 cm^−1^ Raman intensity ratio is associated with volumetric changes in hemoglobin signals.^27^ Since the 1275 cm^−1^ peak intensity remains flat with no significant change over time for any model, this normalized ratio of 1209/1275 likely reflects changes in blood content. Both LPS‐ and RU486‐treated cervices showed an increase in blood signals along the longitudinal labor trajectory (Figure 3a). The D19‐term cervices had higher blood signal values compared to D15‐control, indicating that the increase observed in D19 occurred between D15 and D19 within the term group. Similar trends also appeared for another hemoglobin Raman band at 1545 cm^−1^, previously assigned to blood.^8^, ^27^

**FIGURE 3:**
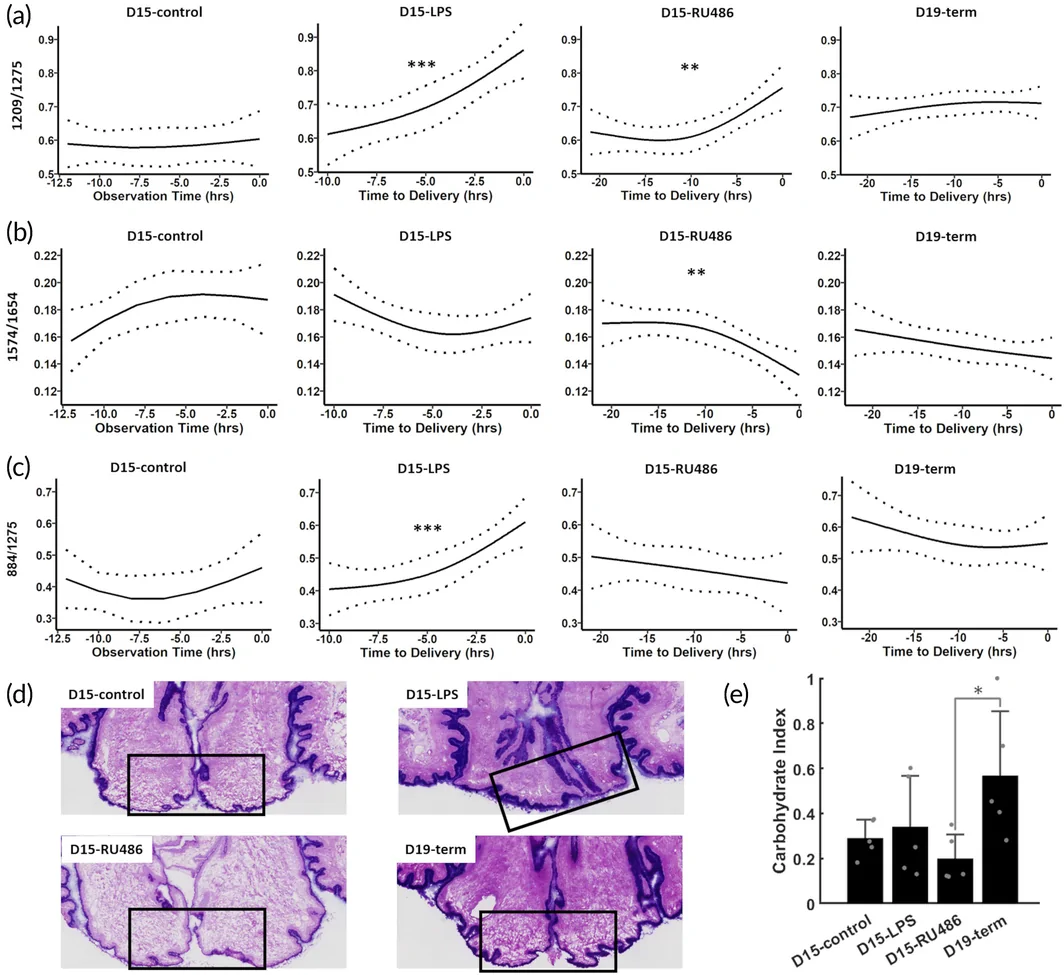
In vivo blood, DNA and carbohydrates spectral markers during preterm labor (PTL). Longitudinal labor trajectory until delivery (0 h) at (a) 1209 cm^−1^/1275 cm^−1^—blood, (b) 1574 cm^−1^/1654 cm^−1^—DNA, and (c) 884 cm^−1^/1275 cm^−1^—carbohydrates plotted for four groups: D15‐control, D15‐LPS, D15‐RU486, and D19‐term (left to right) with 95% confidence intervals (**p < 0.01, ***p < 0.001, significant change assessed by ANOVA) in slope of the trajectory with respect to horizontal (slope = 0 line) (*n* = 10–12 per cohort). D15‐control trajectory for 12 h was found to be unchanged as expected. A significant increase in blood (hemoglobin) was observed for both D15‐LPS and D15‐RU486—PTL models. Similar changes were seen for another blood Raman marker at 1545 cm^−1^. In vivo DNA‐spectral markers decreased during RU486 induced PTL and carbohydrates markers increased during LPS‐induced PTL. (d) AB‐PAS‐stained images (4× magnification) for D15‐control, D15‐LPS, D15‐RU486, and D19‐term. (e) Integrated density of the purple area mostly located along the epithelium (edges), calculated from the AB‐PAS–stained images from the area marked in the black rectangle, shows carbohydrates increasing in D15‐LPS during labor to level up to D19‐term, although changes were not significant. Unexpectedly, D15‐RU486 showed a decrease in carbohydrates. Kruskal–Wallis test was used here for the data with non‐normal distribution requiring a nonparametric test. Bar graph values are expressed as mean ± SEM (*n* = 5 per cohort).

### 2.5 Nuclei decrease in density during labor

Nucleic acids can be identified using two specific peaks (782 and 1574 cm^−1^) in a tissue‐like complex system.^23^ This study focused on the 1574 cm^−1^ region due to interference from the Raman probe below 800 cm^−1^. The 1574 cm^−1^/1654 cm^−1^ ratio revealed a decrease in nucleic acid signal relative to protein close to the delivery in D19‐term and RU486‐treated mice compared with D15‐control (Figure 3b).

### 2.6 Carbohydrates and mucins increase in LPS and decrease in RU486 models

The 884 cm^−1^/1275 cm^−1^ ratio reflects changes in carbohydrates, including glycosaminoglycans (GAGs) and mucins, as the 884 cm^−1^ band corresponds to carbohydrate C–O–C skeletal and C–H deformation modes,^28^, ^29^ while the 1275 cm^−1^ band remained stable across all models. This ratio therefore directly represents carbohydrate and/or mucin content. A significant increase in the 884/1275 ratio over time was observed only in the LPS group. Notably, the D19‐term cervix exhibited higher 884/1275 values than the D15 control, indicating an increase between D15 and D19 (Figure 3c). Although a similar trend was observed at another carbohydrate‐associated band (1367 cm^−1^), blood interference led us to use the 884 cm^−1^ band for longitudinal analysis. In contrast, RU486‐treated mice showed a decreasing trend in carbohydrate/mucin‐related bands, although the change was not statistically significant. To validate these observations, Alcian blue–PAS staining was performed (Figure 3d), and a carbohydrate index based on segmented epithelial thickness‐to‐gray‐value ratio was quantified (Figure 3e). Ex vivo analysis mirrored the in vivo results, showing a modest, non‐significant carbohydrate increase in LPS‐treated cervices and significantly reduced carbohydrate levels in RU486‐treated samples compared with D19‐term. These findings challenge the prior hypothesis that RU486 represents an accelerated model of D19‐term labor.^18^

### 2.7 Ex vivo imaging analysis of the cervix corroborated the in vivo RS findings

Figure 4 shows SHG images of cervices excised ~3 h before delivery, demonstrating collagen fibers clumped together and highly dispersed, creating significantly larger pore structures in RU486 mice compared with the other three models. TPEF images from stained elastin fibers showed a statistical decrease in elastin fiber density in RU486‐treated cervix tissues, compared with D15‐control and LPS‐treated groups, especially in the subepithelial stroma. Blood vessel fractional area analyzed from CD31‐stained sections showed an increasing trend for labor models compared with D15‐control, although the changes were not statistically significant. Perhaps ex vivo stained cervices, which reflect results from collapsed blood vessels observed in a limited field of view, do not fully represent in vivo blood flow. Nuclei fractional area calculated from DAPI‐stained images showed a significant decrease in RU486 and D19 models compared with D15‐control, consistent with increasing tissue hydration and cell separation.

**FIGURE 4:**
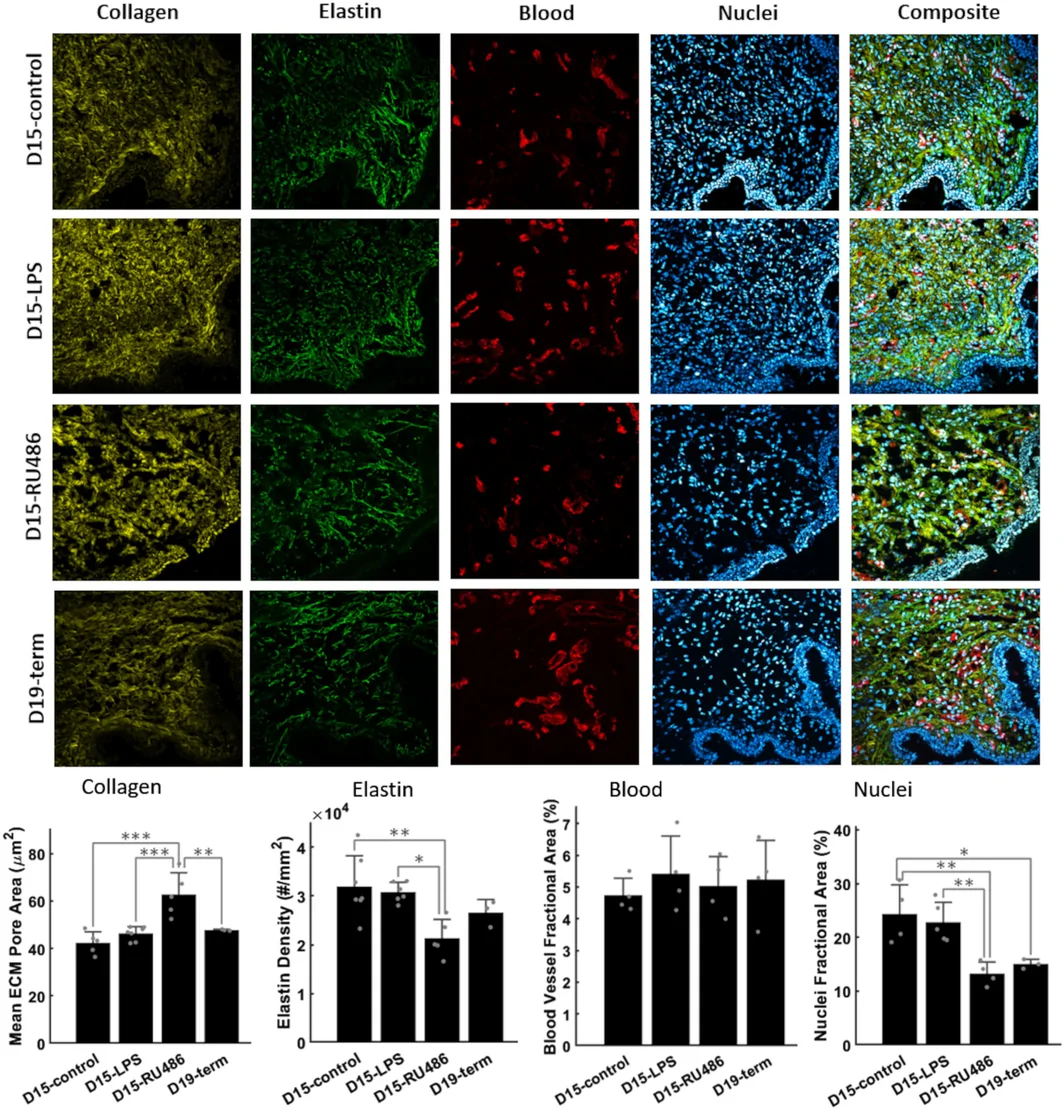
Ex vivo images show the spatial distribution of biochemicals in the ectocervix during labor. Column‐1: SHG images showing the changes in collagen fibers' alignment. RU486 collagen changes suggest degradation, where fibers are clumped together and dispersed creating a more porous structure, whereas D15‐control and D15‐LPS were more alike. Column‐2: Elastin immunostained images showing the changes in the density/length of the fibers. Column‐3: CD31 stained images for blood vessels showing an increase in vessel diameter (area) during labor in D15‐LPS and D19‐term models compared with D15‐control. Column‐4: DAPI‐stained images of nuclei showing a decrease in density during labor in D15‐RU486 and D19‐term models compared with D15‐control, whereas D15‐control and D15‐LPS appear similar. Column‐5: Composite image of all biochemical components. Ex vivo images of 10 μm cryo‐sectioned cervix tissues were recorded with a home‐built multimodal microscope system. The quantification parameters from columns 1 to 4 are represented as a bar graph in the bottom row. Bar graph values are expressed as mean ± SEM (*n* ≥ 4 per cohort) where *p < 0.05, **p < 0.01, ***p < 0.001 significant change assessed by ANOVA.

### 2.8 Lipid molecules such as PGE are essential for the onset of (term and preterm) labor

The 1301 cm^−1^/1275 cm^−1^ Raman ratio reveals changes in lipid derivatives and precursor molecules.^30^ All three labor models (LPS, RU486, D19‐term) show an increasing trend compared with the D15‐control, indicating that lipid molecules play a crucial role in labor onset, regardless of etiology (Figure 5a). A Raman intensity ratio at 877 cm^−1^/855 cm^−1^ was analyzed without conclusive spectral assignment. This ratio showed a significant increase only for LPS over the time course post injection; however, an opposite trend can be seen for RU486 (Figure 5b), suggesting an informative RS ratio that can differentiate varying etiologies of cervical remodeling with labor. Although the biochemical assignment for these bands is complex, this ratio is highly specific to LPS and may help identify inflammation/infection‐induced labor.

**FIGURE 5:**
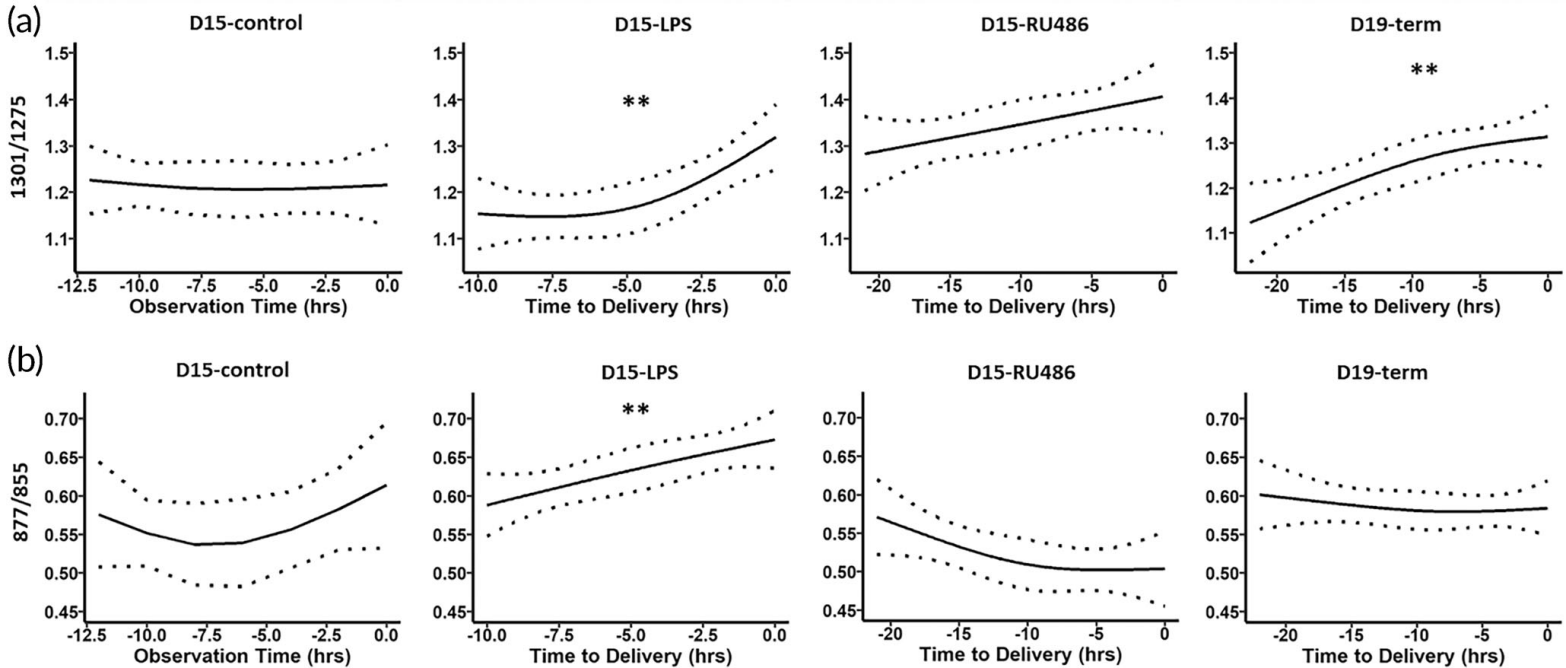
In vivo Raman signatures significantly change as the cervix prepares for delivery. (a) Longitudinal labor trajectory until delivery (0 h) at 1301 cm^−1^/1275 cm^−1^ plotted for four groups (left to right: D15‐control, D15‐LPS, D15‐RU486, D19‐term) with 95% confidence interval (**p < 0.01, significant change assessed by ANOVA in slope of the trajectory with respect to horizontal (slope = 0 line)). 1301/1275 depicts changes in lipid‐like molecules, PGE_2_, or PGE_2_ precursors. D15‐control trajectory for 12 h was unchanged, as expected. (b) Longitudinal labor trajectory until delivery (0 h) at 877 cm^−1^/855 cm^−1^ plotted for four groups (left to right: D15‐control, D15‐LPS, D15‐RU486, D19‐term) with 95% confidence intervals (**p < 0.01, significant change in slope of the trajectory with respect to horizontal (slope = 0 line)). 877/855 depicts changes in hydroxyproline/proline ratio along with changes in other amino acids (*n* = 10–12 per cohort).

### 2.9 A multivariate model with RS peak ratios demonstrates classification between the three labor models

Key Raman bands were used to develop a principal component analysis (PCA)‐based linear discriminant analysis (LDA) model. This included all the bands discussed previously, along with additional bands that are difficult to assign to a single component but exhibited trends relevant to labor trajectories: 817, 855, 877, 884, 961, 1080, 1160, 1209, 1251, 1275, 1301, 1321, 1367, 1402, 1447, 1521, 1545, 1574, and 1654 cm^−1^. The multivariate model successfully separated all three labor cohorts. The 3D LDA scatter plot clearly illustrates the separation between all three labor models (Figure 6). The model achieved an overall accuracy of 85.6%, with few misclassifications (Figure 6c).

**FIGURE 6:**
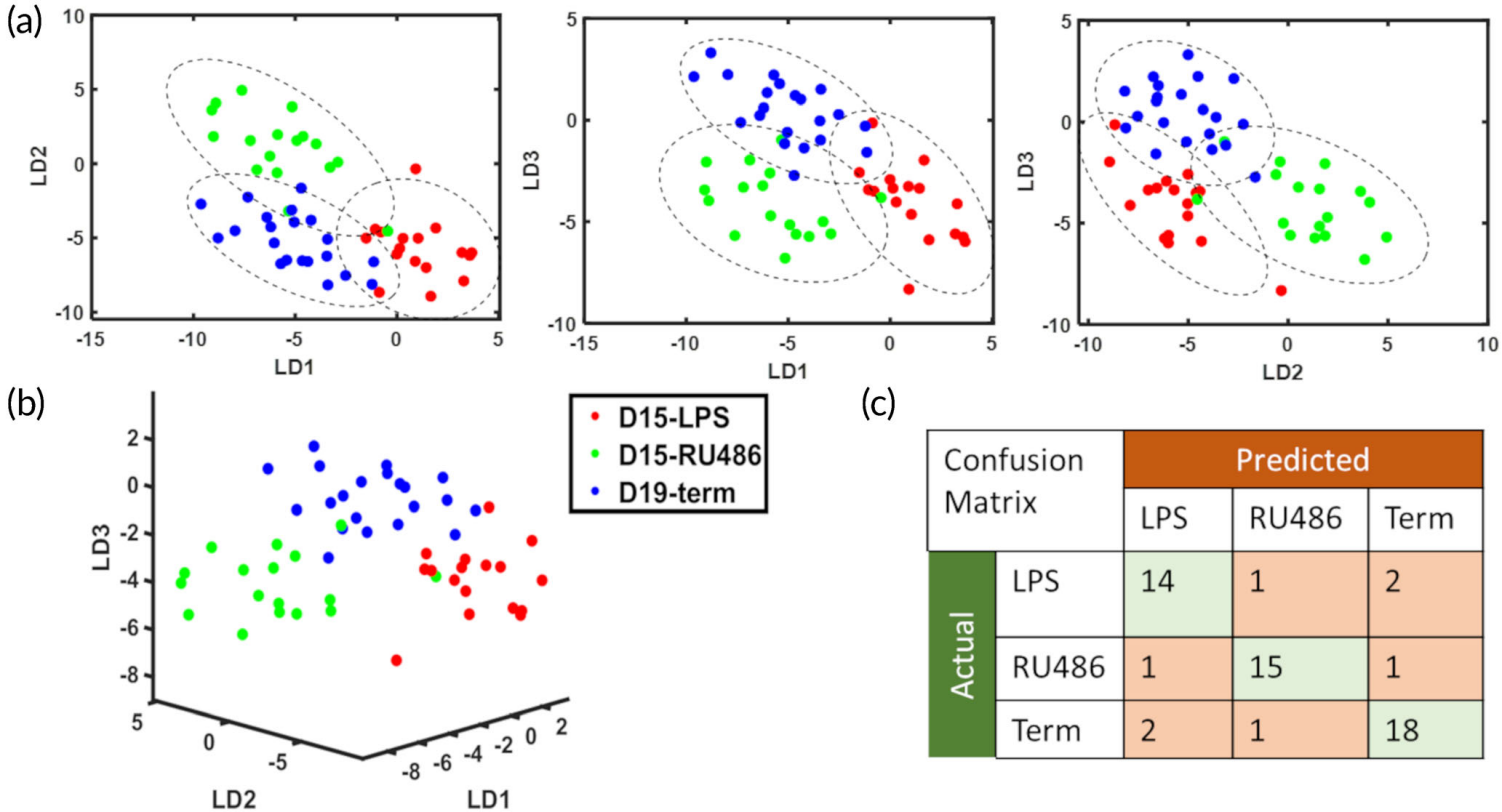
The combination of in vivo Raman bands significantly differentiates D19‐term from preterm labor (D15‐LPS and D15‐RU486). (a) 2D‐ LD1 vs. LD2, LD1 vs. LD3, and LD2 vs. LD3 plots show a reliable separation of D19‐term from D15‐LPS and D15‐RU486 with 90% confidence ellipsoids highlighting group separations. (b) 3D‐linear discriminant (LD) analysis on selective Raman features plot depicts significant separation between all three groups. (c) related confusion matrix depicting overall accuracy of 85.6%.

## 3 DISCUSSION

Cervical remodeling is essential for successful parturition. Therefore, understanding the physiological and biochemical changes underlying cervical maturation and labor onset is critical for improved prevention, early detection, and management of PTL. Since cervical remodeling pathways vary by labor etiology, a non‐invasive tool identifying these changes before or during labor could reveal unexplored physiology and offer valuable diagnostic insights.

The cervix comprises collagen and elastin fibers, smooth muscle, matricellular proteins, glycans, water, and cells, whose dynamic/temporal interactions define labor onset. This complexity is difficult to capture using traditional ex vivo staining or single‐component analyses. This study utilized in vivo RS to compare mice induced into preterm delivery using LPS or RU486 with untreated mice delivering at term. These PTL models mirror premature cervical remodeling in women, enabling controlled investigation of distinct remodeling pathways. Distinct spectral signatures were observed in LPS, RU486, and term mice, showcasing the ability of in vivo RS to detect biochemical changes during labor and differentiate between inflammation/infection‐driven and progesterone withdrawal–driven remodeling. Importantly, in vivo molecular changes preceding delivery correlated with ex vivo imaging and biomechanical measurements.

Previous studies established the feasibility of in vivo RS for monitoring cervical remodeling during normal pregnancy in mice and women and demonstrated biochemical changes associated with gestational progression and labor.^8^, ^15^, ^32^ The present study advances beyond these feasibility investigations in several important ways. First, the customized ball lens probe enabled depth‐selective interrogation of the murine cervix while minimizing spectral contamination from surrounding adipose tissue, a limitation of previous mouse studies.^32^ Second, this work establishes the first biochemically validated Raman biomarkers of distinct PTL pathways through pairing of in vivo Raman measurements with ex vivo biomechanics, SHG/TPEF imaging, and histological analysis. Additionally, the current work demonstrates that inflammatory‐ and progesterone withdrawal‐induced PTL exhibit distinct biochemical remodeling trajectories that can be differentiated non‐invasively in vivo. This moves RS beyond pregnancy monitoring toward a mechanistically validated cervical phenotyping platform.

The biochemical framework of the ECM critically governs cervical viscoelastic mechanical properties.^33^ We observed a significant decrease in the Raman band at 817 cm^−1^ (C‐C backbone of ECM proteins), distinguishing PTL from TL (Figure 2a). This optically detectable decrease occurred in PTL models before delivery, with values approaching those of term cervices. The C‐C backbone structure (817 cm^−1^) accommodates tensile stress imparted to the tissue through helix stretching and molecular kink straightening, thereby reflecting tensile stress‐induced structural changes, in which stretching reduces vibration energy.^25^, ^26^, ^34^, ^35^ Intensity decreases correlate with protein backbone strain and deformation during stretching, directly affecting tissue mechanical strength. In PTL, this biochemical change coincided with rapid cervical dilation in response to labor, catching up with D19‐term cervices (Figure 2c). Typically, cervical ripening and dilation at term occur before the onset of labor.^36^ Concurrent ripening and dilation (influenced by uterine contractions) in PTL likely impose abnormal tension on the premature cervix, as evidenced by abrupt 817 cm^−1^ reductions. Enhanced MMP/ADAMTS protease activity in PTL may drive accelerated collagen remodeling, bypassing physiological term ripening.^18^, ^37^ Specifically, MMPs such as MMP8, MMP4, ADAMTS1, and ADAMTS4 are upregulated in premature cervical ripening and dilation induced by RU486 and LPS.^18^, ^37^

Interestingly, cervical ripening induced by RU486 shows distinct ECM protein disruption compared with LPS and TL models. The amide III/I ratio significantly decreased in RU486‐treated mice during labor but remained unchanged in LPS and TL (Figure 2b). Since amide I reflects backbone structure and amide III relates to side chains, their combined assessment provides insights into ECM integrity^31^, ^38^ and their relative intensities are also linked to collagen fiber orientation.^39^ Furthermore, crosslinks and hydrogen bonds between the C=O and N‐H groups of adjacent collagen molecules are critical for fiber stability, and the decrease in the amide III/I ratio in RU486 cervices suggests rupture of these bonds between collagen triple helices, leading to disordered protein structure.^40^, ^41^ This hypothesis aligns with the disrupted collagen fibers observed in SHG images of the RU486 model, resulting in increased porosity (Figure 4). Ex vivo biomechanical tests also support this, showing lower equilibrium elastic energy in RU486‐treated cervices compared with D19‐term (Figure 2e). It is plausible that the reduced equilibrium elastic energy in RU486‐treated cervices arises from the reduced elastin fiber density and a more porous ECM.^32^ Prior studies report increased pore size and a 54% rise in pore fractional area in RU486‐treated versus term mice, indicating collagen disorganization.^18^, ^42^ This disruption of collagen fibers may also be linked to lower carbohydrate and mucin content in RU486 mice (Figure 3c–e), as carbohydrates are believed to act as secondary links that stabilize collagen fibrils.^33^

Although collagen and elastin both play a load‐bearing mechanical role in the cervix,^33^ quantitative measures of elastin have not been reported extensively. During normal pregnancy, elastin undergoes progressive ultrastructural reorganization, transitioning from long, densely packed fibers to shorter, loosely arranged structures by gestational D18 in untreated mice.^43^ Notably, composite SHG and immunofluorescence images demonstrate that the elastin fibers align with collagen fibers (Figure 4). Progesterone primarily regulates collagen organization, whereas estrogen governs elastin processing and assembly. By term, densely packed elastin fibers become fragmented and disorganized.^43^ In RU486‐treated cervices, the disruption of collagen fibers due to progesterone receptor blockade (Figure 2b) may divert mechanical stress onto elastin fibers, potentially causing further disruption in elastin fiber packing (Figure 4).

Pregnancy‐induced alterations in vascular function are conserved between humans and mice.^44^ Recent studies in humans demonstrated that Raman bands related to blood significantly increase throughout pregnancy.^8^, ^15^ We noted a similar increase in blood signal, at 1209 and 1545 cm^−1^, in both LPS and RU486 models during labor, while D19‐term cervices exhibited elevated blood levels compared to D15‐control (Figure 3a). This increase likely reflects vasodilation and expanded cervical vasculature. Notably, blood signals surge within 5 h post‐LPS treatment, suggesting that this increase is primarily associated with vasodilation during this short timeframe. The prostaglandin moiety PGE_2_, a vasodilator produced by both cyclooxygenase isoforms, Ptgs1 (RU486) and Ptgs2 (LPS), which are upregulated during premature cervical ripening, drives blood signal increases in PTL.^18^ However, in TL, blood signals rise steadily from D15 to D19 (Figure 3a), whereas PGE_2_/lipid‐like molecules only surge during active labor. This temporal disconnect, corroborated by ex vivo vascular imaging (Figure 4), suggests that additional signaling molecules contribute to term remodeling. An increase in vascular area in the D19‐term model may be due to both an increase in blood vessels from VEGF^45^ and vasodilation, which is associated with distinct mechanisms, including changes in relaxin hormone levels.^46^, ^47^

Nuclei density decreases as pregnancy progresses in both mice and humans, correlating with cervical enlargement and expansion of the extracellular space.^36^, ^48^ All laboring mice show reduced nuclei density, but this trend is most pronounced in RU486‐treated and TL models (Figure 4). The diminished nucleic acid signal at 1574 cm^−1^ in RU486‐treated cervices likely reflects increased tissue wet weight and porosity (Figure 3b), which drive cervical volume expansion akin to D19‐term cervices.^18^, ^36^

Carbohydrates, including GAGs and mucins, contribute to cervical remodeling by modulating collagen organization, facilitating cell adhesion, and recruiting immune cells.^49^, ^50^ In LPS‐treated cervices, Raman signatures at 884 cm^−1^ (indicative of carbohydrates/mucins) increased pre‐delivery, approaching term (D19‐term) levels, likely driven by inflammation‐induced glycan upregulation (Figure 3c–e). This aligns with studies linking elevated glycan levels to innate immune activation during LPS‐ or HSV‐2‐mediated cervical injury.^50^, ^51^ In contrast, RU486‐treated cervices exhibited reduced total carbohydrates, corroborated by diminished AB‐PAS staining (Figure 3c–e). While sulfated‐to‐non‐sulfated GAG ratios shift toward non‐sulfated GAGs (increased hyaluronic acid) during pregnancy,^50^ methodological limitations prevent Raman or AB‐PAS from distinguishing specific GAG subtypes. Thus, the observed decrease in RU486 models may reflect loss of other specific carbohydrates and mucins. This decrease could also be explained by an increase in the volume of the cervix, which is likely to be accompanied by a decrease in the concentration of biochemical components present.^18^, ^36^ To date, the role of glycans in cervical remodeling remains unclear; for instance, decorin‐deficient mice (devoid of this dominant cervical GAG) show normal parturition^36^ and small proteoglycans decline before labor, suggesting redundancy or compensatory mechanisms.^52^ Further investigations are needed to gain insights into the role of overall proteoglycan levels during pregnancy.

Lipids and lipid‐like molecules (e.g., PGE_2_) stimulate proinflammatory responses.^18^, ^53^ The Raman signature at 1301 cm^−1^, associated with lipids, increased during TL and in both PTL (Figure 5). This aligns with clinical observations where laboring women exhibit elevated lipid‐like signatures, correlating with rising levels of lipid‐based inflammatory mediators.^15^ Macrophages are key drivers of ripening‐phase remodeling and perform distinct functions, including synthesizing lipid derivatives.^36^, ^53^, ^54^ The increase in lipid bands could be associated with increased production of lipid mediators including PGE_2_ precursors synthesized by Ptgs1 and Ptgs2.^18^ While some biomolecules exhibited similar trends in term and PTL, their temporal patterns differ. The D19‐term mice demonstrate comparatively less remodeling during labor leading up to delivery, with significant changes occurring in the ripening period from D15 to D19. Notably, the lipid‐like feature at 1301 cm^−1^ shows significant increase during labor, but not between D15 and D19 (Figure 5). This observation aligns with the fact that neither Ptgs1 nor Ptgs2 is upregulated during term ripening; rather, their upregulation is observed during labor and postpartum repair phase.^18^, ^55^

Further, the Raman intensity ratio at 877 cm^−1^/855 cm^−1^ was significantly enhanced for LPS‐treated mice while showing the reverse trend in RU486‐treated mice (Figure 5). This ratio, previously linked to hydroxyproline/proline ratio in collagen, indicates an increase in non‐enzymatic glycation of collagen due to a supramolecular rearrangement.^56^ However, this spectral region also has contributions from lipids, carbohydrates, and nuclei. Despite the challenges in assigning specific biomarkers, the change in this ratio suggests unique intermolecular interactions during infection/inflammation‐induced cervical ripening that are distinct from those in term and RU486‐induced ripening (Figure 5). The integration of these relevant Raman bands via a multivariate classification algorithm (PCA‐LDA) improved the segregation of term, LPS‐ and RU486‐induced labors with an overall accuracy of 86% (Figure 6). This indicates that the longitudinal trajectories of in vivo spectral markers specific to LPS and RU486 treatment reflect distinct ripening mechanisms in the cervix. Given the limited sample size, not all Raman spectral variables were used to avoid overfitting. With a larger dataset, employing more variables and sophisticated algorithms could further enhance model performance and accuracy.

Importantly, the present study builds upon prior clinical studies that demonstrated safe longitudinal cervical Raman measurements during pregnancy and labor using clinically compatible fiber‐optic Raman probes in more than 90 pregnant participants without adverse events.^8^, ^15^ Furthermore, a speculum‐free image‐guided Raman probe was developed to improve patient comfort and facilitate measurements during labor, demonstrating spectral performance comparable to conventional speculum‐based measurements.^57^ While these studies established clinical feasibility and identified gestational biochemical changes in women, the present work provides mechanistic validation of Raman biomarkers through controlled PTL models that cannot be ethically or practically investigated in humans. The ball lens probe was specifically optimized for murine anatomy to enable depth‐selective cervical measurements that minimize adipose tissue interference. Together, these findings establish a foundation for future prospective studies evaluating RS for PTB risk assessment and etiologic stratification, particularly when integrated with existing clinical tools such as cervical length assessment and fetal fibronectin testing.

This study faces several limitations. External factors, including mucus, blood, or suboptimal probe contact, can affect in vivo cervical measurements. Future human studies may incorporate white‐light imaging to identify and remove contaminants, improving data quality.^57^ While ex vivo imaging of fixed tissue provides valuable insight, it does not fully capture dynamic in vivo processes such as blood flow. Emerging in vivo techniques, including probe‐based SHG^58^, Mueller polarimetric colposcopy,^59^ and Doppler ultrasonography,^60^ could address this limitation by enabling real‐time assessment of protein structure and blood flow. To improve clinical utility, future work should focus on implementing real‐time spectral quality control and developing improved prediction algorithms using larger datasets.

## 4 CONCLUSION

Our study uncovers novel insights into the distinct molecular pathways in RU486‐ and LPS‐induced PTL. It provides a longitudinal perspective showing that cervical ripening in PTL is not merely an acceleration of the physiological process seen in TL. The spectral markers and their temporal relationships shed new light on structure–function interactions, advancing our understanding of premature cervical ripening and enabling early detection for timely intervention. Ultimately, this research demonstrates strong potential for clinical translation, especially for in vivo monitoring of cervical remodeling in pregnant women to improve management and neonatal outcomes.

## 5 MATERIALS AND METHODS

Animals: All experiments adhered to the NIH Guide for Care and Use of Laboratory Animals, using protocols approved by Vanderbilt University Medical Center's Institutional Animal Care and Use Committee (IACUC; protocol no. M1700074‐01). Animal experiments are reported in accordance with the ARRIVE 2.0 guidelines, with relevant information regarding animal characteristics, experimental groups and controls, procedures, sample sizes, outcome measures, and statistical analyses provided throughout the Methods and figure legends. CD‐1 wild‐type mice (Jackson Laboratory) were housed with a 12‐h dark/light cycle. Gestation D1 was determined by the presence of a post‐copulatory plug, and mice typically delivered on D19.5. Four groups were studied: (i) LPS‐induced PTB group generated by intraperitoneal injection of LPS (055:B5, Sigma, St. Louis, MO; 100 μg in 0.1 mL PBS) at 8 am on D15; (ii) progesterone withdrawal‐induced PTB group created by subcutaneous injection of RU486 (mifepristone, Sigma; 150 μg in a mixture of ethanol and sesame oil) at 8 am on D15; (iii) pregnant D19 CD‐1 mice as the term delivery group; and (iv) pregnant D15 CD‐1 mice injected with PBS at 8 am as vehicle controls for LPS‐ and RU486‐treated mice.

Raman spectroscopy: A customized ball lens fiber‐optic Raman probe (~2.1 mm diameter, EMVision) was designed for in vivo transvaginal measurements in the mouse cervix. The probe geometry was selected to accommodate the small dimensions of the pregnant mouse cervix (~2–4 mm in diameter) and vaginal canal while minimizing signal contamination from surrounding adipose tissue.^61^ A sapphire ball lens (2 mm diameter) was incorporated to provide compact optical focusing, mechanical robustness, and minimal Raman or fluorescence background from the lens material. Previous Monte Carlo modeling studies demonstrated that ball lens probes improve superficial sampling and depth selectivity through enhanced focusing and collection efficiency, with smaller ball lens diameters favoring shallower tissue interrogation.^62^ The probe was therefore optimized for shallow cervical sampling, and experimental characterization using layered tissue phantoms demonstrated a penetration depth of approximately 500 μm, well below 1 mm, thereby reducing contributions from adjacent adipose tissue. Unlike our previous mouse study,^32^ in vivo cervical spectra acquired using this probe closely matched ex vivo cervical spectra obtained immediately after necropsy (Figure S1b). For all ex vivo analyses, including RS, biomechanical testing, and imaging, the cervix was dissected under a stereomicroscope to remove uterine and vaginal tissues, as previously described.^63^ Raman data were acquired using a portable RS system consisting of an imaging spectrograph (HoloSpec f/1.8i, Kaiser Optical Systems) coupled to a CCD camera (PIXIS: 256BR, Princeton Instruments), a 785‐nm diode laser (Innovative Photonic Solutions), and the ball lens fiber‐optic probe (EMVision). The probe (~2.1 mm diameter) comprised a central delivery fiber surrounded by seven collection fibers (Figure 1a). The laser power at the sample was set to 80 mW. During measurements, all room lights were turned off. For in vivo measurements, animals were anesthetized using isoflurane. The vaginal cavity and cervix were rinsed with saline, and a speculum tube was inserted for visualization. Raman spectra were recorded at 12, 3, 6, and 9 o'clock positions around the ectocervix, with an integration time of 5 s per measurement. Data were collected from 10 to 12 mice for each model, with measurements taken every 2 h post‐treatment (LPS, RU486, or PBS) until the first pup's delivery or for up to 12 h. Similarly, untreated mice were measured every 2 h on gestation D19 until natural delivery.

Ex vivo biomechanical testing: Cervices were excised ~3 h before delivery (based on average cohort delivery timing) for all labor models and on gestation day 15 (D15) evening for WT mice (negative control). Cervical tissue dimensions (height, length, width) were measured, followed by immediate placement in oxygenated Krebs Bicarbonate buffer at 37°C for 10 min to equilibrate before stress‐relaxation testing using a Radnoti Organ Bath System as described previously,^32^ which allows assessment of the viscoelastic behavior of each cervical tissue. Stress–strain relationships in the toe region, elastic region, yield point (indicative of the structural shift point from elastic‐to‐plastic phases), breakpoint (tissue failure under tension), and other mechanical properties were determined.^64^

Ex vivo imaging: Cervices were excised ~3 h before delivery for all labor models and in the evening of gestation D15 for WT mice as negative control and immediately flash‐frozen in liquid nitrogen and stored at −80°C. Flash‐frozen tissues were cryo‐sectioned transversely into 10 μm‐thick sections, then labeled using antibodies against elastin (1:250 dilution in blocker solution) with secondary Alexa fluor goat anti‐rabbit 488 (1:2000), CD31 (1:50 dilution in blocker solution) with secondary Alexa fluor goat anti‐rat 555 (1:1000) and stained with DAPI to locate nuclei. Separately, 7 μm cervical sections were stained with Alcian blue–PAS to visualize glycans and mucopolysaccharides. Staining and imaging were performed at Vanderbilt shared resource facilities using automated whole‐section microscopy at up to 20× magnification.

SHG and TPEF images were acquired using a custom‐built multimodal imaging platform equipped with a photomultiplier tube (GaAsP Amplified PMT, Thorlabs) on the epi‐detection port.^65^, ^66^ Each image was integrated for 7 microseconds/pixel and recorded using a 20× water immersion objective (Olympus XLUMPLFLN, 1.0 NA) with a high spatial sampling density (401 nm/pixel) covering 1024 × 1024 pixels per image. SHG signals were generated using 798 nm and collected using a 400/±40 nm filter. Similarly, TPEF images were obtained from antibody‐labeled sections, using excitation at 750 nm with 460/±40 nm filter for DAPI, 940 nm with 525/±90 nm filter for elastin, and 1040 nm with 625/±40 nm filter for CD31.

Data analysis: Wavenumber calibration for in vivo RS was performed using neon–argon, naphthalene, and acetaminophen standards, while system spectral response was corrected using a National Institute of Standards and Technology–calibrated tungsten lamp. Approximately 10–15% of spectra were excluded due to high adipose contribution surrounding the cervix or excessive noise (including accidental room light). Spectra were smoothed using a Savitzky–Golay filter, baseline‐corrected with the Asymmetric Least Squares method (λ = 6, *p* = 0.00005), and normalized using Standard Normal Variate transformation across the full spectral range.^67^ Ratiometric analysis was employed to compare spectra with varying signal intensities. Temporal trends in Raman peak ratios were modeled using restricted cubic spline regression with three nodes, plotted as mean ± 95% confidence intervals, and analyzed by ANOVA to assess significance of rate changes during labor (*p* < 0.05) using R software. PCA was performed on labor (D19‐term, LPS, RU486) data for dimensionality reduction in MATLAB (MathWorks, Natick, MA). Subsequently, an LDA model was developed based on the PC that accounted for ~95% of the data variance. Model robustness was evaluated using 10‐fold cross‐validation, with 90% of the data used for training and 10% for validation in each iteration.

Ex vivo images were analyzed using ImageJ (Fiji)^68^ and MATLAB. To reduce shot noise, all images were first processed using a 0.5‐pixel Gaussian blur. Collagen fibers in SHG images were extracted using the Steger (Ridge) method in ImageJ (Fiji) and converted into binary fiber masks.^69^, ^70^ These masks were inverted to generate pore masks, followed by application of a watershed transform to separate neighboring pores. Individual stromal pores were identified using Moore‐Neighbor tracing, and the mean pore area was calculated.^70^ Elastin fibers were identified from the TPEF images using the Steger (Ridge) method, counted, and normalized to the total stromal area to obtain elastin fiber density. Nuclei were extracted from DAPI‐TPEF images by thresholding at half of the Otsu threshold. The number of pixels within the resulting binary nuclear mask was divided by the total stromal pixel count to calculate the nuclear (DNA) fractional area. For blood vessel analysis, TPEF images exhibited elastin fluorescence bleed‐through, which was removed using an unsupervised k‐means spectral unmixing algorithm.^71^ Blood vessels were subsequently segmented by thresholding at half of the Otsu threshold, followed by a flood‐fill operation (MATLAB imfill) to fully mask vessel regions. Blood vessel fractional area was calculated by normalizing vessel pixels to the total stromal area. For AB‐PAS–stained cervix sections, the epithelium was segmented and both mean epithelial thickness and mean gray value were quantified. A carbohydrate index was defined as the ratio of thickness‐to‐gray‐value, reflecting both staining extent and intensity, and normalized to a 0–1 scale by division by the maximum index across all samples. Statistical comparisons for all metrics were performed using ANOVA followed by a Tukey–Kramer post‐hoc multiple comparison test, with *p* < 0.05 considered statistically significant.

## AUTHOR CONTRIBUTIONS

**Rekha Gautam:** Conceptualization; formal analysis; methodology; investigation; validation; writing – original draft; writing – review and editing. **Christine M. O'Brien:** Conceptualization; formal analysis; writing – review and editing. **Ezekiel J. Haugen:** Methodology; formal analysis; writing – review and editing; investigation; validation. **Jackson H. Rogers:** Methodology; validation. **Laura E. Butler:** Methodology; formal analysis; writing – review and editing. **Jennifer N. Bateman:** Methodology. **Anna S. Rourke‐Funderburg:** Formal analysis. **Naoko Boatwright:** Methodology; investigation; validation. **Wilson R. Adams:** Methodology; formal analysis; writing – review and editing. **Giju Thomas:** Formal analysis; writing – review and editing; validation. **Jeff Reese:** Conceptualization; methodology; supervision; funding acquisition; resources; writing – review and editing. **Jennifer L. Herington:** Conceptualization; methodology; investigation; supervision; funding acquisition; resources; writing – review and editing. **Anita Mahadevan‐Jansen:** Conceptualization; methodology; investigation; funding acquisition; supervision; resources; writing – review and editing; project administration.

## FUNDING INFORMATION

This study was supported by National Institutes of Health grant HD081121 (JR, AMJ) and National Institutes of Health grant EB033385 (JR, AMJ).

## CONFLICT OF INTEREST STATEMENT

Authors declare that they have no competing interests.

## DECALARATION OF AI USE

ChatGPT (GPT‐5) was used solely for basic grammar, and copy‐editing to improve language clarity, in accordance with Wiley's publication guidelines. The authors reviewed and edited the final text and take full responsibility for its content.

## Supporting information


**TABLE S1.** Observed Raman bands and their assignments.
**FIGURE S1.** Ball‐lens (Φ = 2 mm diameter) probe characterization. (a) The Raman spectra of the fat tissue hidden beneath breast tissue used for depth profiling. The penetration depth of the ball‐lens probe was assessed by examining the exponential decay of the lipid‐to‐breast tissue ratio. This analysis estimated the probing depth to be 500 μm based on the 1/*e* value of the ratio. (b) Raman spectra of the cervix from mice on day 19 of pregnancy were recorded both in vivo and ex vivo using the ball‐lens probe, illustrating a remarkable similarity in cervix spectra under both experimental conditions.
**FIGURE S2.** Raman spectra of selected biomolecules, present in cervix, utilized for relevant bands assignment.

## Data Availability

All data needed to evaluate the conclusions in the paper are present in the paper and/or the Supporting Information file and raw data are available from the authors upon request.
