# Supplementary material for: Biochemical basis of cervical remodeling in preterm and term labor revealed by Raman spectroscopy
Source: Bioeng Transl Med. 2026 Nov 10:e70193. Online ahead of print. doi: 10.1002/btm2.70193 (PMC13750464; doi:10.1002/btm2.70193)
Supplement: Supplementary file 1 [file BTM2-9999-e70193-s001.docx]

Supporting Information

**Biochemical basis of cervical remodeling in preterm and term labor revealed by Raman spectroscopy**

*Rekha Gautam^1,2^*******, Christine M. O’Brien^1,^3, Ezekiel J. Haugen^1^, Jackson H. Rogers^4^, Laura E. Masson^1^, Jennifer N. Bateman^1^, Anna S. Rourke-Funderburg^1^, Naoko Boatwrigh^4^, Wilson R. Adams^1^, Giju Thomas^1^, Jeff Reese^1,4^*‡, Jennifer L. Herington^4^*‡, Anita Mahadevan-Jansen^1^*‡*

^1^Department of Biomedical Engineering, Vanderbilt University, Nashville, TN 37232, USA.

^2^Biophotonics@Tyndall, IPIC, Tyndall National Institute, Lee Maltings Complex, Cork, Ireland

^3^Department of Biomedical Engineering, Washington University in St. Louis, St. Louis, MO, USA

^4^Department of Pediatrics, Vanderbilt University Medical Center, Nashville, TN 37232, USA.

***To whom correspondence may be addressed.**

rekha.gautam@tyndall.ie

jeff.reese@vumc.org, jennifer.l.herington@vumc.org, [anita.mahadevan-jansen@Vanderbilt.Edu](mailto:anita.mahadevan-jansen@Vanderbilt.Edu)

Department of Biomedical Engineering, Vanderbilt University, Nashville, TN 37232, USA

‡These authors contributed equally to this work

**Table of content:**

1. **Figure S1.** Ball-lens (Φ = 2 mm diameter) probe characterization.
2. **Figure S2.** Raman spectra of selected biomolecules, present in cervix, utilized for relevant bands assignment.
3. **Table S1**. Observed Raman bands and their assignments.

Figure S1. Ball-lens (Φ = 2 mm diameter) probe characterization. (A) The Raman spectra of the fat tissue hidden beneath breast tissue used for depth profiling. The penetration depth of the ball-lens probe was assessed by examining the exponential decay of the lipid-to-breast tissue ratio. This analysis estimated the probing depth to be 500 μm based on the 1/e value of the ratio. (B) Raman spectra of the cervix from mice on day 19 of pregnancy were recorded both in vivo and ex vivo using the ball-lens probe, illustrating a remarkable similarity in cervix spectra under both experimental conditions.

Fig. S2. Raman spectra of selected biomolecules, present in cervix, utilized for relevant bands assignment.

Table S1. Observed Raman bands and their assignments.

| Raman shift (cm^–1^) | Spectral assignments |
| --- | --- |
| 1630–1680 | Amide I (C=O stretch coupled to a N-H bending) in proteins; C=C stretch in lipids |
| 1615 | C=C stretch in tyrosine, tryptophan; ν(C_a_=C_b_) of vinyl groups in porphyrin |
| 1574 | Nucleic acid (ring breathing modes in adenine (A), guanine(G)) |
| 1545 | ν(C_β_C_β_) stretch in porphyrin; amide II in proteins |
| 1521 | C=C stretch in carotenoids |
| 1447 | CH_2_, CH_3_ bending in proteins and lipids |
| 1398-1405 | COO^-^ symmetric stretch in amino acids; CH_3_ bending in proteins |
| 1360-1380 | Pyrrole ring symmetric stretch in porphyrin; C-H deformation in carbohydrates |
| 1338 | Nucleic acid (A,G); ν (pyrrole half-ring) symmetric stretch in porphyrin; CH_2_, CH_3_ twisting in proteins |
| 1321 | CH_2_, CH_3_ twist/wag in proteins, lipid; ring vibrations in nucleic acids |
| 1301 | CH_2_ twist/wag/deformation (lipids) |
| 1220-1310 | Amide III (NH in-plane bending and C-N stretching) in proteins |
| 1209 | δ (C_m_H) in-plane deformation in porphyrin |
| 1160 | C-C stretch in carotenoids; C-C/C-N stretch in proteins |
| 1125 | ν(C-C) skeletal of acyl backbone in lipid (trans conformation); C-O stretch in carbohydrates; C-N stretch in proteins |
| 1080 | ν(C-C) skeletal of acyl backbone in lipid (gauche conformation); C-O, C-C stretch in carbohydrates; PO_2_^-^ stretching in nucleic acids |
| 1004 | Symmetric ring breathing mode of phenylalanine |
| 961 | PO_4_^3-^ symmetric stretching; C-C in proteins |
| 938 | C-C stretch α-helix protein, C-O-C glycosides in carbohydrates |
| 920 | C-C stretch of proline ring |
| 884 | C-O-C skeletal and C-H deformation modes in carbohydrates |
| 877 | ν(C-C) hydroxyproline; phospholipids; C-O-C ring carbohydrates |
| 855 | Ring breathing in tyrosine; CC-H deformation (aromatic side chains); C-C stretching in proteins |
| 817 | C-C skeletal mode in structural proteins |
